# Supplementary material for: Effects of Crawling before Walking: Network Interactions and Longitudinal Associations in 7-Year-Old Children
Source: Int J Environ Res Public Health. 2022 May 3;19(9):5561. doi: 10.3390/ijerph19095561 (PMC9100207; doi:10.3390/ijerph19095561)

Supplementary Figure S1. Flow chart of the children included in the study.

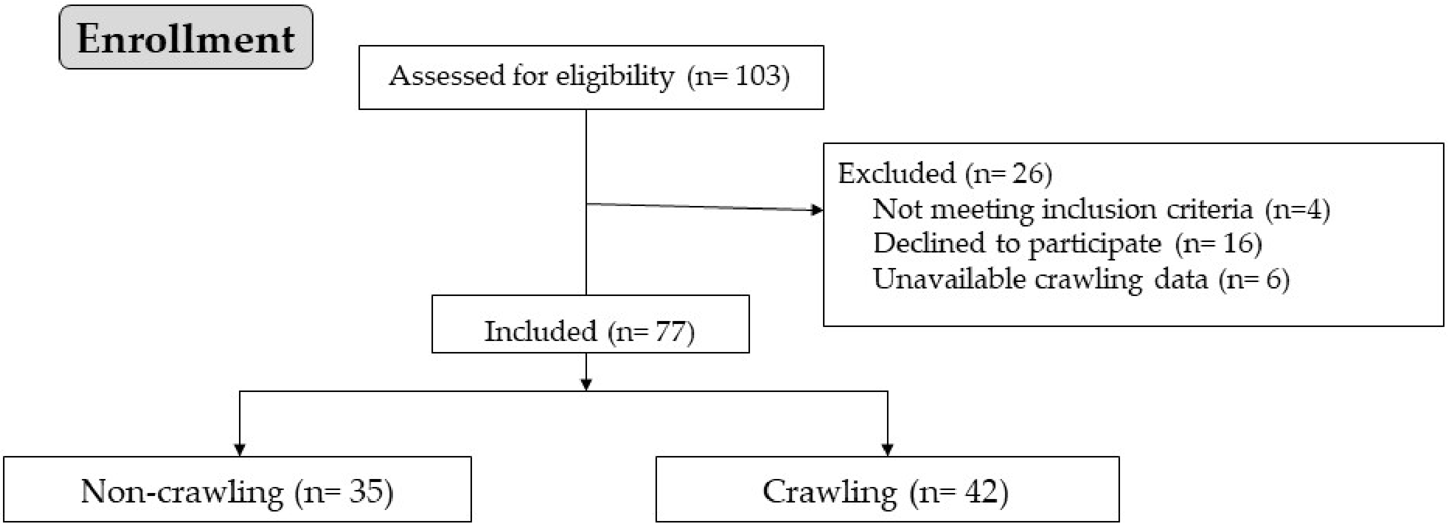

Supplementary Figure S2. Graphs represents overlaid box plots for fat mass %, BMI z–score, fat–to–muscle ratio and systolic and diastolic blood pressure between the non–crawling and the crawling group. *p*-Values are shown on top of each panel.

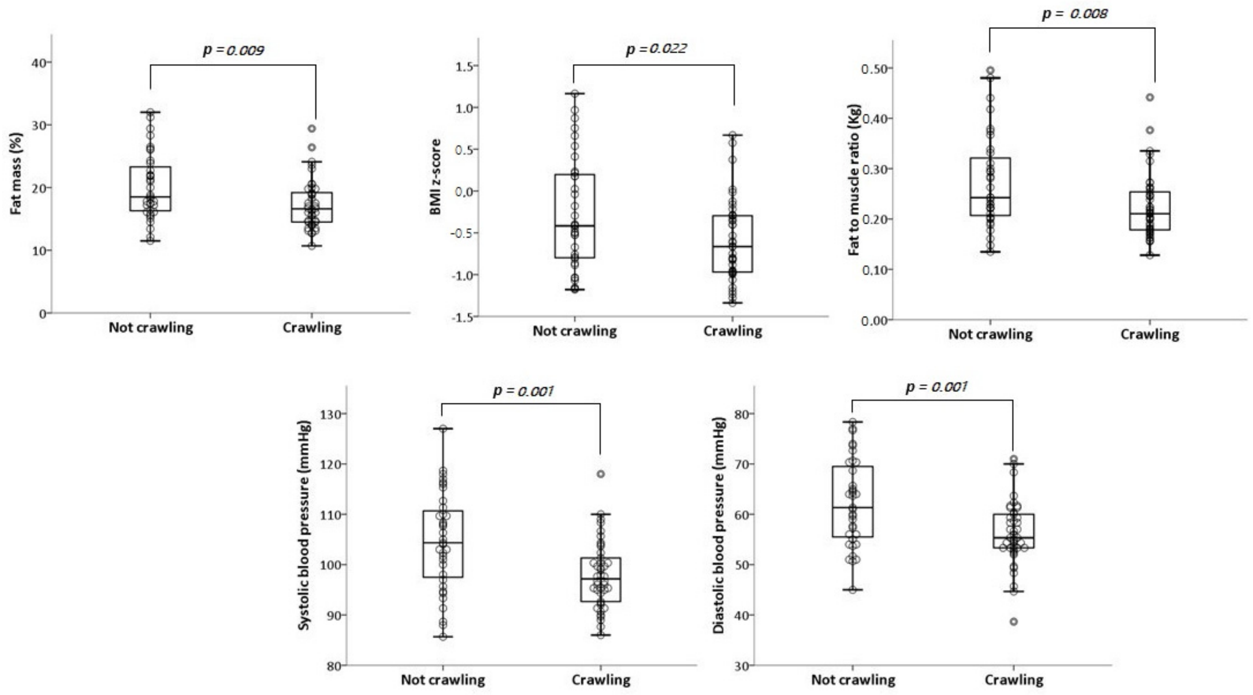

Supplement: Supplementary file 1 [file ijerph-19-05561-s001.zip › ijerph-1662414-supplementary.pdf]
